# Anilin – Addendum: Reevaluierung des BAT-Wertes und Evaluierung einer Schwangerschaftsgruppe zum BAT-Wert: Beurteilungswerte in biologischem Material

**DOI:** 10.34865/bb6253d10_1ad

**Published:** 2025-03-31

**Authors:** Britta Brinkmann, Rüdiger Bartsch, Sandra Michaelsen, Gerlinde Schriever-Schwemmer, Hans Drexler, Andrea Hartwig, MAK Commission

**Affiliations:** 1 Institut für Angewandte Biowissenschaften. Abteilung Lebensmittelchemie und Toxikologie. Karlsruher Institut für Technologie (KIT) Adenauerring 20a, Geb. 50.41 76131 Karlsruhe Deutschland; 2 Friedrich-Alexander-Universität Erlangen-Nürnberg. Institut und Poliklinik für Arbeits-, Sozial- und Umweltmedizin Henkestraße 9–11 91054 Erlangen Deutschland; 3 Ständige Senatskommission zur Prüfung gesundheitsschädlicher Arbeitsstoffe. Deutsche Forschungsgemeinschaft, Kennedyallee 40, 53175 Bonn, Deutschland. Weitere Informationen: Ständige Senatskommission zur Prüfung gesundheitsschädlicher Arbeitsstoffe | DFG

**Keywords:** Anilin, Methämoglobin, Biologischer Arbeitsstoff-Toleranzwert, BAT-Wert, Entwicklungstoxizität, Schwangerschaftsgruppe

## Abstract

The German Senate Commission for the Investigation of Health Hazards of Chemical Compounds in the Work Area (MAK Commission) re-evaluated the data for aniline [62-53-3] to verify the biological tolerance value (BAT value) of 500 μg aniline/l urine and to assign the BAT value to a pregnancy risk group, considering all toxicological end points. Relevant studies were identified from a literature search. The critical effect of aniline exposure in humans is considered to be the formation of methaemoglobin. It must be ensured that the methaemoglobin value remains below 5%, even if the BAT value is maintained. In an experimental study, the methaemoglobin content in human blood rose from 0.72% to an average methaemoglobin value of 1.2% (maximum of 2.07% methaemoglobin) during six hours of exposure to 2 ml aniline/m^3^ (corresponding to the maximum concentration at the workplace, MAK value). With linear extrapolation an excretion of 224 μg aniline/l would be expected at the end of an eight-hour exposure period. Consideration of the increased respiratory volume and the critical methaemoglobin increment results in a concentration of 500 μg aniline (after hydrolysis)/l urine. The BAT value has therefore been confirmed. Due to acute toxic effects, the BAT value must be regarded as the maximum value, i. e. it must be ensured that this value is not exceeded. Sampling should take place at the end of exposure or the end of a shift.

Because the no observed adverse effect concentration (NOAEC) for methaemoglobin content in relation to developmental toxicity in humans is not known, a risk for the unborn child cannot be ruled out, even in cases of compliance with the BAT value. The BAT value is therefore assigned to Pregnancy Risk Group B. As an indication of the prerequisite for an assignment to Pregnancy Risk Group C, a concentration of 30 µg aniline/l urine has been calculated from the lowest value of the natural background range of mean methaemoglobin values in (pregnant) women. At this urinary aniline concentration, damage to the embryo or foetus is unlikely.

**Table:** 

**BAT-Wert (2015)**	**500 μg Anilin (nach Hydrolyse)/l Urin** Ableitung des BAT-Wertes als Höchstwert wegen akut toxischer Effekte
	Probenahmezeitpunkt: Expositionsende bzw. Schichtende
Fruchtschädigende Wirkung (2024)	Gruppe B, Voraussetzung für Gruppe C: 30 µg Anilin (nach Hydrolyse)/l Urin
**BLW (2015)**	**100 μg Anilin (aus Hämoglobin-Konjugat freigesetzt)/l Blut**
	Probenahmezeitpunkt: nach mindestens 3 Monaten Exposition

**MAK-Wert (1983)**	**2 ml/m^3^ ≙** **7,7 mg/m^3^**
Fruchtschädigende Wirkung (2024)	Gruppe B

## Reevaluierung

Im Jahr 2015 wurde für Anilin aus den Daten der Studie von Käfferlein et al. (2014) ein BAT-Wert von 500 μg Anilin (nach Hydrolyse)/l Urin als Höchstwert wegen akut toxischer Effekte abgeleitet (Bolt et al. 2016). Der MAK-Wert von 2 ml/m^3^ (Henschler 1992) wurde im Jahr 2024 der Schwangerschaftsgruppe B zugeordnet (Hartwig und MAK Commission 2025). Da der BAT-Wert nicht in Korrelation zum MAK-Wert abgeleitet wurde, ist die Zuordnung zu einer Schwangerschaftsgruppe für den BAT-Wert zu prüfen.

## Toxikokinetik und Metabolismus

Es werden nur Daten zum Menschen dargestellt. Informationen zur Toxikokinetik beim Tier sind ausführlich bei Greim (2007), Hartwig und MAK Commission (2018) und bei Bolt et al. (2016) beschrieben.

### Halbwertszeit für Methämoglobin

Bei massiven Belastungen durch aromatische Amino- oder Nitroverbindungen mit deutlich erhöhten Methämoglobinwerten (Intoxikationen) beträgt deren Halbwertszeit bis zu 12 Stunden (Bolyai et al. 1972; Leng und Bolt 2008). Die Halbwertszeit des Methämoglobins beträgt nach inhalativer Exposition gegen Anilin bei der Ratte 75 Minuten (Greim 2007; Kim und Carlson 1986).

Aus einer Studie an 19 Personen, die 6 Stunden gegen 2 ml Anilin/m^3^ inhalativ exponiert wurden (Käfferlein et al. 2014), geht hervor, dass die Halbwertszeit für Methämoglobin ca. 3 bis 4 Stunden beträgt, wobei die initiale Halbwertszeit mit ca. 2 Stunden angegeben werden kann. Eine Akkumulation über die Arbeitswoche ist damit nicht zu erwarten.

An trächtigen Ratten wurde gezeigt, dass Anilin die Plazentaschranke passieren kann. Innerhalb einer Stunde nach subkutaner Verabreichung an die Muttertiere (Dosis nicht bekannt) befand sich mehr Anilin im fetalen als im mütterlichen Plasma. Die Halbwertszeit im Blut war mit ca. 1,5 Stunden bei Muttertieren und Feten gleich (Henschler 1990; Maickel und Snodgrass 1973).

## Hintergrundbelastung: Anilin

Bei 856 Nichtrauchenden und 145 Rauchenden wurden für die Nichtrauchenden eine mediane Urinkonzentration von 2,99 µg Anilin/l Urin und ein 95. Perzentil von 14,49 µg Anilin/l Urin (Bereich < 0,1–384) und für Rauchende eine mediane Urinkonzentration von 3,28 µg Anilin/l Urin und ein 95. Perzentil von 13,46 µg Anilin/l Urin (Bereich 0,19–35,71) bestimmt (Kütting et al. 2009). 

Für 81 Nichtrauchende der Allgemeinbevölkerung (48 Frauen, 33 Männer; Alter 20–61 Jahre) wurde der Median mit 1,88 µg Anilin/l, das 95. Perzentil mit 10,9 µg Anilin/l und das Maximum mit 130 µg Anilin/l Urin angegeben (Seidel 2005).

### Schwangere Frauen

In einer Untersuchung von 300 schwangeren Frauen (244 Nichtraucherinnen, 56 Raucherinnen) in Brasilien zur Bestimmung aromatischer Amine im Urin wurden folgende Konzentrationen an Anilin im Urin angegeben: Median: 1,38 µg Anilin/l, 25. Perzentil: 0,89 µg Anilin/l, 75. Perzentil: 2,87 µg Anilin/l, Minimum: 0,48 µg Anilin/l, Maximum: 218,84 µg Anilin/l Urin (Souza et al. 2023).

Bei 171 schwangeren Frauen wurden in den USA im Zeitraum von 2008 bis 2020 im Urin 45 Chemikalien gemessen. In allen Urinproben wurde Anilin nachgewiesen; die Konzentrationen betrugen: geometrischer Mittelwert: 0,81 µg Anilin/l, Median: 0,74 µg Anilin/l, 25. Perzentil: 0,52 µg Anilin/l, 75. Perzentil: 1,1 µg Anilin/l, Minimum: 0,15 µg Anilin/l, Maximum: 34 µg Anilin/l Urin (Choi et al. 2022).

## Hintergrundbelastung: Methämoglobin

Die Hintergrundbelastung mit Methämoglobin in der nicht exponierten Allgemeinbevölkerung stellt sich wie folgt dar: Mittelwert: 0,78 %, Standardabweichung: 0,37 %, Median: 0,85 %, 95. Perzentil: 1,28 % (ACGIH 2006). Als Obergrenze kann 1,5 % Methämoglobin festgesetzt werden. Der physiologische Hintergrundbereich für Methämoglobingehalte wird im Mittel mit < 1 % angegeben. Die Erhöhung des Methämoglobingehaltes beim Menschen über den Wert von 1,5 % hinaus ist als Expositionsmarker anzusehen und zeigt eine Exposition gegen Methämoglobin-Bildner an (Leng und Bolt 2008).

In einer Probandenstudie wurden 9 Frauen und 10 Männer 6 Stunden lang gegen 2 ml Anilin/m^3^ exponiert. Die Originaldaten zur Methämoglobinbildung der 9 Frauen sind in Tabelle 1 dargestellt (Käfferlein 2023). Zu Beginn der Exposition betrug der Mittelwert für Methämoglobin bei den 9 Frauen 0,75 % (Bereich: 0,53–0,97 %). Zusätzlich wurden bei 8 Kontrollpersonen 5 Tage lang die Methämoglobingehalte bestimmt. Der Mittelwert betrug 0,58 ± 0,15 % (Bereich 0,2–1 %) und das 95. Perzentil 0,8 %. Der Schwankungsbereich innerhalb eines Tages wird mit 21 %, der innerhalb von 5 Tagen mit 29 % angegeben (Käfferlein et al. 2014). Da die Probanden nur während eines Sechstels der Expositionszeit mit einem Atemvolumen von 30 l/min auf einem Fahrradergometer belastet wurden, entspricht dies einem durchschnittlichen Atemvolumen von 12,5 l/min, wenn ein Ruheatemvolumen von 9 l/min zu Grunde gelegt wird. Die Daten zeigen, dass es nach 6-stündiger inhalativer Exposition gegen 2 ml/m^3^, hochgerechnet auf 8 Stunden und unter Berücksichtigung des erhöhten Atemvolumens am Arbeitsplatz (21 l/min), bei 8 von 9 Frauen zu einer Überschreitung von 1,5 % Methämoglobin kommen würde. Die Urinkonzentrationen betrugen nach 6 Stunden Exposition gegen 2 ml Anilin/m^3^ bei den 9 Frauen im Mittel ca. 150 µg Anilin/l (siehe Tabelle 2).

**Tab. 1:** Methämoglobingehalte [%] von 9 Probandinnen bei 6-stündiger Exposition gegen 2 ml Anilin/m^3^ (Käfferlein 2023)

Probandin^a)^	Zeitpunkt [h]										MetHb^d)^	MetHb^e)^
	0	2^c)^	4^c)^	6^c)^	7	8	10	12	24	48		
1 langsam	0,70	1,20	1,70	2,07	–	1,40	0,97	1,00	0,60	0,83	2,52	**3,76**
2 schnell	0,70	1,05	1,13	1,27	–	0,87	0,70	0,83	0,50	0,90	1,46	**1,97**
3 langsam	0,97	0,90	0,95	1,30	1,30	0,77	1,00	1,03	1,10	0,77	1,41	**1,71**
4 langsam	0,85	0,90	0,97	1,33	1,00	0,57	0,83	0,87	0,50	0,57	1,49	**1,93**
5 schnell	0,80	0,83	1,00	1,07	0,80	0,67	0,73	0,63	0,53	0,45	1,16	**1,40**
6 langsam	0,63	0,80	0,77	0,90	0,70	0,67	0,75	0,60	0,63	0,53	0,99	**1,23**
7 langsam	0,93	0,93	1,07	1,30	1,00	1,00	0,87	0,70	0,73	0,67	1,42	**1,75**
8 langsam	0,53	0,73	0,87	1,03	0,80	0,83	0,80	0,70	0,67	0,60	1,20	**1,65**
9 langsam	0,60	0,80	0,97	1,03	0,77	0,60	0,73	0,70	0,60	0,40	1,18	**1,57**
**MW**	**0,75**	**0,82**	**1,05**	**1,26**	**0,91**	**0,82**	**0,82**	**0,79**	** 0,65**	** 0,64**	**1,43**	**1,89**
**MW (Inkrement^b)^)**				**0,51**	**0,16**	**0,07**	**0,07**	**0,04**	**–0,1**	**–0,11**		

^a)^ Acetyliererstatus

^b)^ Inkrement: Differenz des Methämoglobingehalts zum Zeitpunkt 0 h

^c)^ unter Exposition gegen 2 ml Anilin/m^3^

^d)^ linear hochgerechnet auf 8-Stunden-Exposition aus Inkrement 0–6 h

^e)^ erhöhtes Atemvolumen für das Inkrement berücksichtigt

MetHb: Methämoglobin; MW: Mittelwert

**Tab. 2:** Anilinkonzentration im Urin und Methämoglobininkrement [%] im Blut von 9 Frauen, die über 6 Stunden gegen 2 ml Anilin/m^3^ exponiert waren (Käfferlein 2023, 2024)

Probandin^a)^	Anilin [µg/l Urin]	Anilin [µg/l Urin]	MetHb-Inkrement	Anilin [µg/l Urin]	MetHb-Inkrement	Anilin [µg/l Urin]	MetHb-Inkrement
	vor Exposition	nach 2 h Exposition		nach 4 h Exposition		nach 6 h Exposition	
1 langsam	6,67	118,35	0,5	256,21	1	343,49	1,37
2 schnell	7,48	53,68	0,35	174,03	0,43	107,33	0,57
3 langsam	4,86	53,37	-0,07	129,33	-0,02	163,27	0,33
4 langsam	4,50	45,77	0,05	70,05	0,12	79,5	0,48
5 schnell	5,67	57,17	0,03	72,29	0,2	92,31	0,27
6 langsam	1,51	51,52	0,17	117,62	0,14	95,82	0,27
7 langsam	1,24	50,76	0	101,5	0,14	173,97	0,37
8 langsam	7,32	147,5	0,2	183,77	0,34	199,12	0,5
9 langsam	5,95	53,24	0,2	110,08	0,37	96,96	0,43
**Mittelwert**	**5,02**	** 70,18**	** 0,16**	**134,99**	** 0,3**	**150,2**	**0,51**

^a)^ Acetyliererstatus

h: Stunden; MetHb: Methämoglobin

In einer Longitudinalstudie wurde der Einfluss von nitrathaltigem Trinkwasser auf den Methämoglobingehalt bei bis zu 357 schwangeren Frauen untersucht (siehe Tabelle 3). Die Mittelwerte lagen je nach Schwangerschaftswoche im Bereich von 0,39–0,74 % Methämoglobin (Maximalwert 3,6 %). Die Studie fand eine geringe Anzahl von Schwangeren (2 bis 5) mit einem Methämoglobingehalt über der von Wright et al. (1999) als physiologische Obergrenze definierten Grenze von 2 %. Der Mittelwert des Methämoglobingehaltes nimmt während der Schwangerschaft ab, auch nach Berücksichtigung einiger Kovariaten. Diskutiert wird der Einfluss von Vitaminen auf die niedrigen Methämoglobin-Werte. Ob es zu Komplikationen während der Schwangerschaft oder zu Effekten bei den Neugeborenen kam, wird nicht beschrieben (Manassaram et al. 2010).

**Tab. 3:** Zusammenfassende Darstellung der Hintergrundwerte zu Methämoglobin

Kollektiv	Methämoglobin [%] Mittelwert ± SD	Bereich	Schwankungsbereich 21 % (innerhalb eines Tages)/ 29 % (innerhalb von 5 Tagen)	Literatur
9 ♀	0,75	0,53–0,97	0,16/0,22 %	Käfferlein et al. 2014
1 ♀	0,27	–	–	
1 ♀	0,03	–	–	
8 Kontrollpersonen (keine weiteren Angaben) (Nichtrauchende, 5 Tage)	0,58 ± 0,15 0,8 (95. Perzentil)	0,2–1	0,12/0,17 %	
schwangere Frauen:				Manassaram et al. 2010
357 (Erstuntersuchung)	0,74 ± 0,48	0,1–2,2; n = 2^a)^	0,16/0,22 %	
317 (20. SSWo)	0,67 ± 0,52	0,1–3,6; n = 3^a)^	0,14/0,19 %	
316 (28. SSWo)	0,58 ± 0,46	0,1–2,1; n = 2^a)^	0,12/0,17 %	
304 (36. SSWo)	0,51 ± 0,46	0,1–2,2; n = 2^a)^	0,11/0,15 %	
300 (am Tag der Geburt)	0,42 ± 0,47	0,1–2,3; n = 2^a)^	0,09/0,12 %	
295 (2-4 Wochen nach Geburt)	0,39 ± 0,51	0,1–3,0; n = 5^a)^	0,08/0,11 %	
10 Schwangere mit komplikationsloser Schwangerschaft	1,3 ± 0,9	0,4–2,8; n = 3^a)^	0,27/0,38 %	Tabacova et al. 1997

^a)^ mit > 2 % Methämoglobin

SD: Standardabweichung; SSWo: Schwangerschaftswoche

## Polymorphismus der Methämoglobin-Reduktase

Für die Reduktion von Methämoglobin zu Hämoglobin ist die lösliche NAD(P)H-abhängige Cytochrom b5-Reduktase 3 im Zytoplasma verantwortlich, die in Erythrozyten exprimiert wird. Als Elektronenakzeptor ist das am endoplasmatischen Retikulum membrangebundene Cytochrom b5-Typ A (Gen: *CYB5A*) zusätzlich für die Reaktion notwendig (Percy und Lappin 2008). Bei der seltenen kongenitalen Methämoglobinämie liegen Mutationen im *CYB5R3*-Gen vor. Bisher wurde über mehr als 80 verschiedene krankheitsverursachende Varianten im *CYB5R3*-Gen berichtet. Basierend auf der Schwere des Enzymdefekts, werden zwei verschiedene Phänotypen der autosomal rezessiv vererbbaren Methämoglobinämie unterschieden. Beim Typ I führt eine Missense-Variante nur in den Erythrozyten zu einem instabilen Enzym. Als Symptom tritt eine von Geburt an bestehende Zyanose auf, die verbunden ist mit leichten Beschwerden, wie Kopfschmerzen, Mattigkeit und Belastungsdyspnoe und in der Regel gut toleriert wird. Es treten Methämoglobinspiegel bis 30 % auf. Die meisten Patienten sind symptomfrei. Der Typ I-Phänotyp tritt mit einer Häufigkeit von 1:1000 in einigen isolierten Populationen auf. Beim Typ II kommt es zu einer niedrigen Expressionsrate oder niedrigen Aktivität der Cytochrom b5-Reduktase 3 in allen Geweben, was zu einem veränderten Lipidstoffwechsel und neurologischen Beeinträchtigungen führt. Der Methämoglobinspiegel liegt im Bereich von 8 bis 40 %. Die Typ II-Methämoglobinämie weist aufgrund von neurologischen Störungen (Opisthotonus, axiale Hypotonie, variable Dystonie, choreoathetoide Bewegungen), Mikrozephalie und retardiertem Wachstum einen deutlich schwereren Krankheitsverlauf auf. Diese Effekte treten ab dem 9. Lebensmonat auf. Die Sterblichkeit bis zum Erwachsenenalter ist sehr hoch. Ein Mangel an *CYB5R* in bestimmten Bevölkerungsgruppen der USA oder Russland ist nachgewiesen. Ein Selektionsvorteil des afrikaspezifischen Polymorphismus *CYB5R3-*Thr117Ser, der bei 46 % der Afroamerikaner nachgewiesen wurde, bietet vermutlich Schutz vor einer Malariainfektion (Iolascon et al. 2021).

## Reevaluierung des BAT-Wertes

Als kritischer Effekt der Anilinwirkung beim Menschen wird die Methämoglobinbildung angesehen. Die Erhöhung des Methämoglobingehaltes beim Menschen über den Wert von 1,5 % hinaus ist im Sinne des Referenzwertekonzeptes als Expositionsmarker anzusehen und nicht als gesundheitsbasiert zu verstehen. Eine Erhöhung über 1,5 % zeigt eine Exposition gegen Methämoglobinbildner an. Gesundheitsschädliche Effekte durch Methämoglobin sind bei gesunden Erwachsenen bis zu einem Methämoglobingehalt von 5 % nicht zu erwarten (Leng und Bolt 2008).

Die Probandenstudie (9 Frauen und 10 Männer) von Käfferlein et al. (2014) zeigt, dass nach sechsstündiger Exposition gegen 2 ml Anilin/m^3^ (inhalativ; dermaler Kontakt war weitestgehend ausgeschlossen) der Methämoglobingehalt im Blut von 0,72 % auf einen mittleren Methämoglobinwert von 1,2 % ansteigt. Als höchster individueller Methämoglobinwert wurde 2,07 % gemessen. Der Höchstwert des durch Anilin induzierten Methämoglobininkrements betrug 1,35 % (2,07 %–0,72 % = 1,35 %). Es wurde kein Plateau erreicht, so dass eine lineare Extrapolation auf acht Stunden erfolgt. Das Inkrement des Höchstwertes beträgt damit nach acht Stunden 1,8 % (1,35 % × 8/6). Unter Berücksichtigung des erhöhten Atemminutenvolumens am Arbeitsplatz (10 m^3^) ergibt sich ein Methämoglobininkrement von 3 % (21/12,5 l/min = 1,68; 1,8 % × 1,68 = 3 %). Es ist sicherzustellen, dass auch im Einzelfall bei Einhaltung des BAT-Wertes der Methämoglobinwert von 5 % unterschritten bleibt. Das gefundene 8-Stunden-Inkrement ist unter experimentellen Bedingungen einer Anilinexposition in Höhe des MAK-Wertes um den Faktor 1,33 (4 %:3 %) kleiner als ein bei Berücksichtigung der Hintergrundbelastung von < 1 % Methämoglobin maximal zulässiges Methämoglobininkrement von 4 %. Der Mittelwert der Anilinausscheidung betrug nach 6-stündiger Exposition 168 μg/l Urin; am Ende einer 8-stündigen Exposition wäre daher eine Ausscheidung von 224 μg/l zu erwarten. Unter Berücksichtigung des erhöhten Atemvolumens (21/12,5 l/min = 1,68; 224 µg/l × 1,68) ergibt sich eine Konzentration von 376 µg Anilin/l Urin. Legt man den o. a. Faktor von 1,33 zugrunde, so wäre eine Ausscheidung von 500,5 μg Anilin/l Urin zu erwarten. Dieser unter Berücksichtigung des erhöhten Atemvolumens ermittelte Wert

**bestätigt den BAT-Wert von 500 μg Anilin (nach Hydrolyse)/l Urin.**

Der BAT-Wert bezieht sich auf einen Methämoglobinwert von 5 % und ist als Höchstwert anzusehen, d. h. es muss sichergestellt werden, dass dieser Wert nicht überschritten wird. Probenahme ist am Expositionsende bzw. Schichtende.

## Fruchtschädigende Wirkung

### Mensch

#### Methämoglobinbildung als empfindlichster Endpunkt

Bei 61 schwangeren Frauen wurden die Methämoglobinwerte bestimmt. Diese waren unabhängig vom Alter der Mutter, vom Gestationsalter und vom Raucherstatus. Die Methämoglobinwerte lagen bei den 10 Frauen mit normal verlaufender Schwangerschaft zwischen 0,4 und 2,8 %. Insgesamt lagen 64 % der Methämoglobinwerte über der von den Autoren angegebenen physiologischen Obergrenze von 2 %. Komplikationen in der Schwangerschaft wie Anämie (< 10 g Hämoglobin/dl), vorzeitige Entbindung, Blutvergiftung, Proteinurie, Bluthochdruck und Präeklampsie wurden bei erhöhten Methämoglobinwerten bis zu 11,2 % beobachtet. Für die Effekte werden Zellschädigungen, erhöhte Lipidperoxidation und eine Abnahme der antioxidativen Reserven der Mütter verantwortlich gemacht. Die Autoren führen aus, dass die erhöhten Methämoglobinwerte auf eine hohe Exposition gegen Nitrit und Nitrat, sowie weitere Methämoglobinbildner hinweisen (Tabacova et al. 1997). 

In einer weiteren Studie dieser Arbeitsgruppe wurde bei 51 Mutter-Kind-Paaren am Tag der Geburt im Blut der Mutter und im Nabelschnurblut der Methämoglobingehalt und die antioxidative Kapazität bestimmt. Anlass der Studie waren hohe Konzentrationen an NO_2_ in der Umgebungsluft (Mittelwert 23,1 µg/m^3^, Peaks bis 238,5 µg/m^3^) und hohe Konzentrationen an Nitrat im Trinkwasser und Gemüse. Es erfolgte eine Auswertung nach Geburtsgewicht (normal und niedrig (< 2500 g)), Geburt vor der 37. Schwangerschaftswoche und „fetalem distress“. Der Anteil an Raucherinnen betrug 37 %, 8 Mütter hatten während der Schwangerschaft eine Anämie. Bei 36 Müttern verlief die Schwangerschaft ohne Komplikationen, mit normalen Geburtsgewichten, bei 6 Müttern trat eine Frühgeburt auf, bei 7 Neugeborenen war trotz normaler Schwangerschaftsdauer das Geburtsgewicht niedrig, ein Neugeborenes lag in Steißlage und ein Neugeborenes kam durch Kaiserschnitt auf die Welt. 55 % der Methämoglobinwerte im mütterlichen Blut waren oberhalb von 2 % und 47 % der Nabelschnurblutwerte über 2,8 %. Die Methämoglobinwerte im mütterlichen Blut und im Nabelschnurblut waren höher bei denjenigen mit nicht normalen Geburtsergebnissen. Die Methämoglobinwerte der Mütter mit Frühgeburten und mit fetaler Notlage waren etwa doppelt so hoch wie bei Müttern mit normaler Geburt. Die mittleren Methämoglobinwerte im Nabelschnurblut waren bei Frühgeburten viermal so hoch wie bei Normalgeburten, mit einem Höchstwert von 34 %. Der mittlere Methämoglobinwert bei Neugeborenen mit niedrigem Geburtsgewicht war etwa 1,5-mal so hoch wie bei Neugeborenen mit normalem Geburtsgewicht, und der höchste gemessene Wert lag bei 16 %. Rauchen während der Schwangerschaft war nicht mit erhöhten Methämoglobinwerten weder im mütterlichen noch im Nabelschnurblut assoziiert, obwohl das mittlere Geburtsgewicht der Neugeborenen von Raucherinnen niedriger war als bei denjenigen von Nichtraucherinnen (3046 g gegenüber 3197 g). Raucherinnen hatten auch einen höheren Anteil an Frühgeburten (12 % bzw. 6 %). Es zeigte sich ein statistisch signifikant erniedrigte antioxidative Kapazität bei erhöhtem Methämoglobingehalt im mütterlichen und kindlichem Blut (Tabacova et al. 1998).

In einer Umweltstudie von Mohorovic (2003) wird der Zusammenhang zwischen der SO_2_-Umgebungskonzentration und den Methämoglobinwerten bei 260 schwangeren Frauen untersucht. Der Bereich der Methämoglobingehalte kann aus einer Abbildung mit 1,2 bis 2 g/l angegeben werden (Mohorovic 2003). Da keine detaillierten Ergebnisse der Methämoglobinwerte angegeben werden, wird die Studie nicht berücksichtigt.

### Unterschiedliche Empfindlichkeit zwischen Mensch und Ratte und Feten/Neugeborenen

Das fetale Hämoglobin ist leichter oxidierbar als das von Erwachsenen. Die Aktivität der Methämoglobin-Reduktase, die für die Regeneration des funktionstüchtigen Häms aus Methämoglobin verantwortlich ist, zeigt deutliche Speziesunterschiede und ist altersabhängig (Klimmek et al. 1988; Power et al. 2007; Rockwood et al. 2003). Die Aktivität der NADH-abhängigen Reduktase ist in Erythrozyten von Ratten und Mäusen im Vergleich zu denen des Menschen 5- bzw. 10-mal höher (EU 2004; Hartwig und MAK Commission 2017; Smith 1986). In einer Untersuchung an verschiedenen Spezies an adulten Tieren und Neugeborenen sowie auch beim Menschen (3 Erwachsene, 2 Neugeborene) war die Methämoglobin-Reduktase-Aktivität beim erwachsenen Menschen ca. doppelt so hoch wie bei den Neugeborenen. Bei Ratten (3 adulte, 16 neugeborene) und Mäusen (4 adulte, 8 neugeborene) waren die Aktivitäten bei den neugeborenen deutlich höher als bei den adulten Tieren (Lo und Agar 1986). Beim Menschen reagieren aufgrund der niedrigeren Methämoglobin-Reduktase-Aktivität daher Neugeborene auf Methämoglobinbildner deutlich empfindlicher als Erwachsene (Power et al. 2007).

Methämoglobinbildung, -rückbildung und Erythrozytentoxizität bilden einen komplexen, mehrstufigen Prozess. Die Verfügbarkeit antioxidativer Substanzen spielt dabei eine wichtige Rolle (Pauluhn 2004). In Erythrozyten sind die Aktivitäten der Glutathionreduktase beim Menschen etwa 5,5-mal und die der Katalase etwa 2,5-mal so hoch wie bei Ratten (Godin und Garnett 1992). 

## Evaluierung einer Schwangerschaftsgruppe zum BAT-Wert

Untersuchungen zu Effekten bei Neugeborenen, die im Mutterleib gegen einen Methämoglobinspiegel von bis zu 5 % exponiert waren, liegen nicht vor. Methämoglobinspiegel bei Schwangeren werden bis zu 2 % als physiologisch angesehen. Das fetale Hämoglobin wird leichter zu Methämoglobin oxidiert als das von Erwachsenen. Weiterhin ist die Methämoglobin-Reduktase-Aktivität bei Erwachsenen ungefähr doppelt so hoch wie bei Neugeborenen. Aufgrund beider Effekte reagieren Neugeborene deutlich empfindlicher als Erwachsene auf Methämoglobinbildner. Die Aktivität der Methämoglobin-Reduktase ist in den Erythrozyten von Ratten und Mäusen etwa 5- bis 10-mal so hoch wie beim Menschen. Humane Erythrozyten reagieren daher empfindlicher auf Methämoglobinbildner, da sie das Methämoglobin deutlich langsamer reduzieren. Die Bewertung der reproduktionstoxischen Wirkung sollte daher nicht aufgrund von tierexperimentellen Daten erfolgen.

Der von Käfferlein et al. (2014) gefundene individuelle Höchstwert betrug nach 6-stündiger inhalativer Exposition gegen 2 ml Anilin/m^3^ 2,07 % Methämoglobin. Wie bereits beschrieben, wäre das Methämoglobininkrement durch Anilin bei einem erhöhten Atemminutenvolumen von 21 l/min 3 %. Es wird darauf hingewiesen, dass bei Schwangeren das Atemminutenvolumen um etwa 20–50 % nach Ende des ersten Trimenons zunimmt. Es liegt damit aber nicht höher als das angenommene Atemminutenvolumen von 21 l/min bei körperlicher Arbeit.

Da die NOAEC (no observed adverse effect concentration) für den Methämoglobingehalt in Bezug auf die entwicklungstoxische Wirkung beim Menschen nicht bekannt ist und der Foetus deutlich empfindlicher als der Erwachsene reagiert, kann eine Gefährdung des Ungeborenen beim BAT-Wert von 500 µg Anilin/l Urin nicht ausgeschlossen werden. Deshalb erfolgt eine 

**Zuordnung des BAT-Werts von Anilin zu Schwangerschaftsgruppe B.**

Unterstützend wird auf die Zuordnung von Dichlormethan und Kohlenmonoxid in Schwangerschaftsgruppe B in Bezug auf einen CO-Hb-Wert von 5 % hingewiesen.

### Voraussetzung für Zuordnung zu Schwangerschaftsgruppe C

**Abb. 1:**
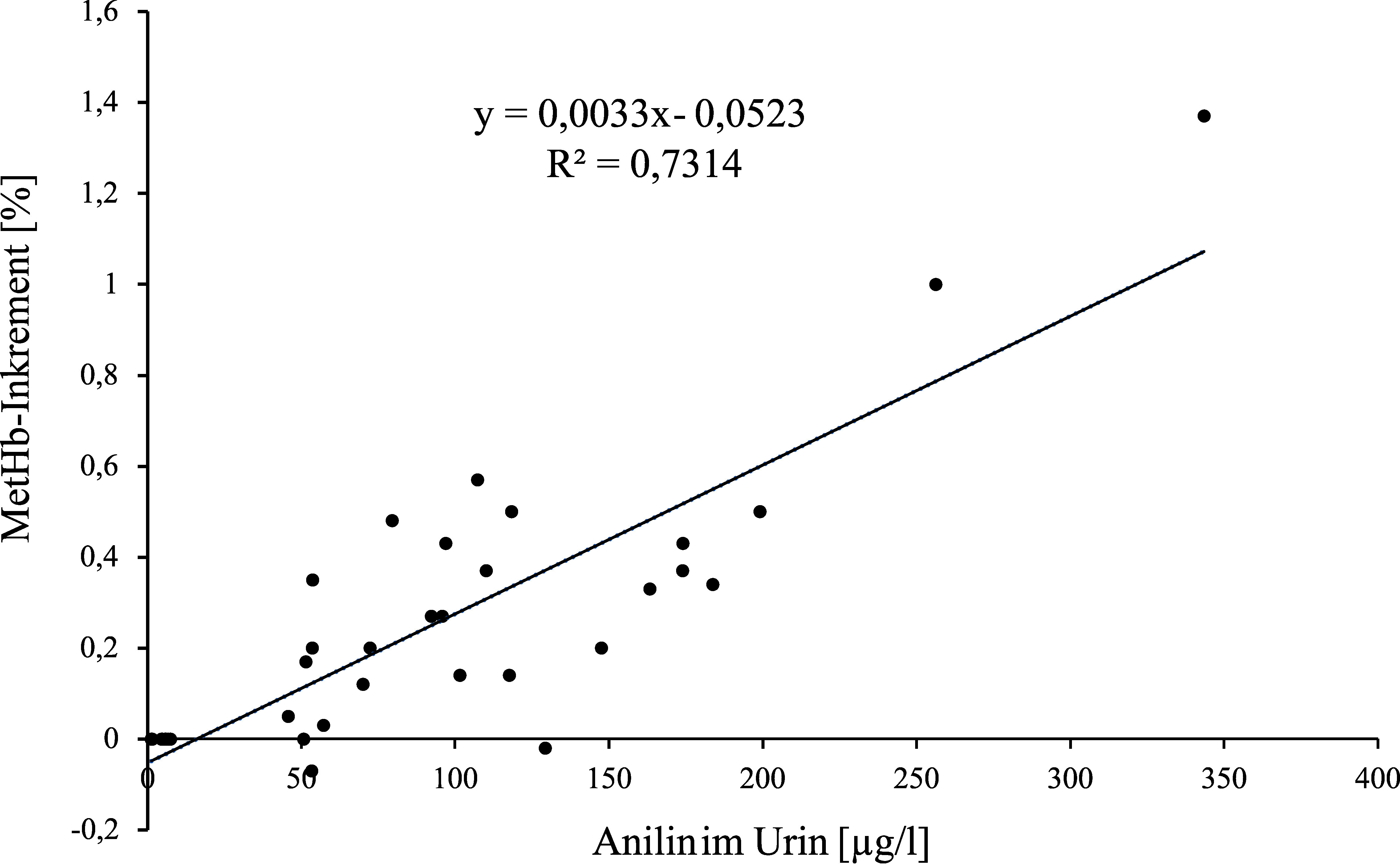
Aus den Einzelwerten der Anilinkonzentration im Urin und den Methämoglobininkrementen der 9 Frauen nach 2-, 4- und 6-stündiger Exposition gegen 2 ml Anilin/m^3^ ermittelte Regressionsgleichung

Die mittleren physiologischen Hintergrundwerte für den prozentualen Anteil an Methämoglobin liegen für Frauen unter 1 %, im Bereich von 0,03 bis 0,97 %. Für schwangere Frauen werden mittlere Hintergrundwerte von 0,39 bis 1,3 % Methämoglobin (Bereich 0,1–3,6 %) angegeben (siehe Tabelle 3). Die Analyse des prozentualen Methämoglobinwerts (MetHb%) bei Schwangeren zeigt einen erheblichen Schwankungsbereich. Dieser Schwankungsbereich beinhaltet jedoch auch die Messungenauigkeit, so dass der tatsächliche Schwankungsbereich der Methämoglobinwerte wahrscheinlich geringer ausfällt. In der Studie von Käfferlein et al. (2014) wird ein Schwankungsbereich innerhalb eines Tages von 21 % und innerhalb von 5 Tagen von 29 % angegeben. Werden diese Schwankungsbereiche auf die Mittelwerte der Methämoglobinwerte bei (schwangeren) Frauen übertragen (siehe Tabelle 3), würden sich Inkremente von minimal 0,08 bis maximal 0,38 % Methämoglobin über den Tag bzw. über 5 Tage ergeben. Bei einer Anilinkonzentration im Urin, die ein Methämoglobininkrement in der natürlichen Schwankungsbreite der Methämoglobingehalte bewirkt, sollte keine Beeinträchtigung des ungeborenen Lebens zu erwarten sein. Für die Bestimmung dieser Anilinkonzentrationen wird die aus den Mittelwerten und/oder den Einzelwerten der Anilinkonzentration im Urin und den Methämoglobininkrementen der 9 Frauen nach 2-, 4- und 6-stündiger Exposition ermittelte Regressionsanalyse verwendet (siehe Abbildung 1 und 2). 

**Abb. 2:**
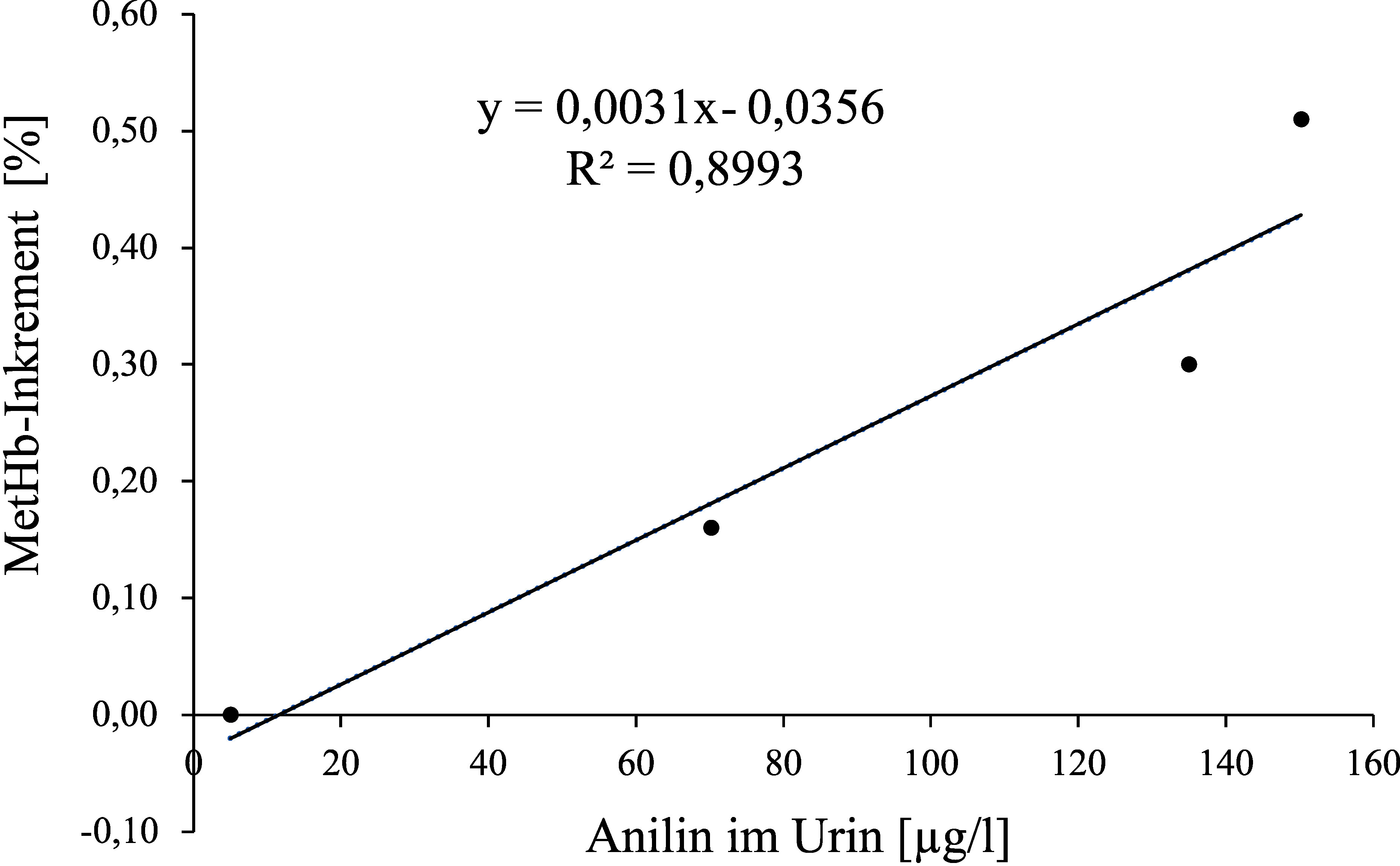
Aus den Mittelwerten der Anilinkonzentration im Urin und den Methämoglobininkrementen der 9 Frauen nach 2-, 4- und 6-stündiger Exposition gegen 2 ml Anilin/m^3^ ermittelte Regressionsgleichung

Methämoglobininkrementen von 0,08 und 0,38 % würden Anilinkonzentrationen von 37,29 und 134,06 µg/l Urin (berechnet aus der Regressionsgleichung basierend auf den Mittelwerten: y = 0,0031x–0,0356 (r² = 0,8993)) oder 40,1 und 131 µg/l Urin (berechnet aus der Regressionsgleichung basierend auf den Einzelwerten: y = 0,0033x–0,0523 (r² = 0,7314)) entsprechen. Da der Fetus auf Methämoglobinbildner wesentlich empfindlicher reagiert als der Erwachsene, wird der unterste Wert des Methämoglobininkrements für die Ableitung der Konzentration als Voraussetzung für Schwangerschaftsgruppe C verwendet.

Als **Voraussetzung für die Zuordnung zu Schwangerschaftsgruppe C** wird eine Konzentration von

**30 µg Anilin/l Urin**

abgeleitet. Bei dieser Konzentration ist eine fruchtschädigende Wirkung nicht anzunehmen.

## References

[ref_CQRB7WVS] American Conference of Governmental Industrial Hygienists ACGIH (2006). Methemoglobin inducers.

[ref_MEF4EJF5] Bolt H. M., Leng G., Drexler H., Hartwig A., MAK Commission (2016). Addendum zu Anilin. BAT Value Documentation in German language. MAK Collect Occup Health Saf.

[ref_74ZKCXDX] Bolyai J. Z., Smith R. P., Gray C. T. (1972). Ascorbic acid and chemically induced methemoglobinemias. Toxicol Appl Pharmacol.

[ref_CWCSRTMK] Choi Giehae, Kuiper Jordan R., Bennett Deborah H., Barrett Emily S., Bastain Theresa M., Breton Carrie V., Chinthakindi Sridhar, Dunlop Anne L., Farzan Shohreh F., Herbstman Julie B., Karagas Margaret R., Marsit Carmen J., Meeker John D., Morello-Frosch Rachel, O’Connor Thomas G., Pellizzari Edo D., Romano Megan E., Sathyanarayana Sheela, Schantz Susan, Schmidt Rebecca J., Watkins Deborah J., Zhu Hongkai, Kannan Kurunthachalam, Buckley Jessie P., Woodruff Tracey J., program collaborators for Environmental influences on Child Health Outcomes (2022). Exposure to melamine and its derivatives and aromatic amines among pregnant women in the United States: the ECHO program. Chemosphere.

[ref_HBETU82B] European Union EU (2004). European Union Summary Risk Assessment Report. Anilin. CAS No. 62-53-3, EINECS No. 200-539-3.

[ref_K7JJDAPL] Godin D. V., Garnett M. E. (1992). Species-related variations in tissue antioxidant status – I. Differences in antioxidant enzyme profiles. Comp Biochem Physiol B.

[ref_R522RAVG] Greim H (2007).

[ref_NH4S56MR] Hartwig A., MAK Commission (2017). N-Methylanilin. MAK Value Documentation in German language. MAK Collect Occup Health Saf.

[ref_X9HTVYHQ] Hartwig A., MAK Commission (2018). Anilin. MAK Value Documentation in German language. MAK Collect Occup Health Saf.

[ref_IFWBQHGL] Hartwig A, MAK Commission (2025). Anilin. MAK-Begründung, Nachtrag. MAK Collect Occup Health Saf.

[ref_3L6XW2P9] Henschler D (1990).

[ref_PIWUZFRV] Henschler D (1992).

[ref_SGXGPJLJ] Iolascon Achille, Bianchi Paola, Andolfo Immacolata, Russo Roberta, Barcellini Wilma, Fermo Elisa, Toldi Gergely, Ghirardello Stefano, Rees Davis, Van Wijk Richard, Kattamis Antonis, Gallagher Patrick G., Roy Noemi, Taher Ali, Mohty Razan, Kulozik Andreas, De Franceschi Lucia, Gambale Antonella, De Montalembert Mariane, Forni Gian Luca, Harteveld Cornelis L., Prchal Josef, SWG of red cell and iron of EHA and EuroBloodNet (2021). Recommendations for diagnosis and treatment of methemoglobinemia. Am J Hematol.

[ref_RBHGURGI] Käfferlein H. U. (2023). Email: Anilin-Daten zur Methämoglobin Bildung.

[ref_9PWW9YXV] Käfferlein H. U. (2024). Email: Anilin-Daten zur Urinkonzentration.

[ref_PE8BCV7B] Käfferlein Heiko Udo, Broding Horst Christoph, Bünger Jürgen, Jettkant Birger, Koslitz Stephan, Lehnert Martin, Marek Eike Maximilian, Blaszkewicz Meinolf, Monsé Christian, Weiss Tobias, Brüning Thomas (2014). Human exposure to airborne aniline and formation of methemoglobin: a contribution to occupational exposure limits. Arch Toxicol.

[ref_YI3I7YI8] Kim Y. C., Carlson G. P. (1986). The effect of an unusual workshift on chemical toxicity. II. Studies on the exposure of rats to aniline. Fundam Appl Toxicol.

[ref_99BRSQBI] Klimmek R., Krettek C., Werner H. W. (1988). Ferrihaemoglobin formation by amyl nitrite and sodium nitrite in different species in vivo and in vitro. Arch Toxicol.

[ref_C4MD3RN6] Kütting Birgitta, Göen Thomas, Schwegler Ursula, Fromme Hermann, Uter Wolfgang, Angerer Jürgen, Drexler Hans (2009). Monoarylamines in the general population – a cross-sectional population-based study including 1004 Bavarian subjects. Int J Hyg Environ Health.

[ref_LGMJIV2K] Leng G., Bolt H.M., Drexler H., Greim H (2008).

[ref_DEAM87GD] Lo S. Chun-Lap, Agar N. S. (1986). NADH-methemoglobin reductase activity in the erythrocytes of newborn and adult mammals. Experientia.

[ref_6JZCQFIC] Maickel R. P., Snodgrass W. R. (1973). Physicochemical factors in maternal-fetal distribution of drugs. Toxicol Appl Pharmacol.

[ref_Z7SIMUZL] Manassaram Deana M., Backer Lorraine C., Messing Rita, Fleming Lora E., Luke Barbara, Monteilh Carolyn P. (2010). Nitrates in drinking water and methemoglobin levels in pregnancy: a longitudinal study. Environ Health.

[ref_NLS6D3NZ] Mohorovic Lucijan (2003). The level of maternal methemoglobin during pregnancy in an air-polluted environment. Environ Health Perspect.

[ref_DLCTLU29] Pauluhn Jürgen (2004). Subacute inhalation toxicity of aniline in rats: analysis of time-dependence and concentration-dependence of hematotoxic and splenic effects. Toxicol Sci.

[ref_5WYZHDPS] Percy Melanie J., Lappin Terry R. (2008). Recessive congenital methaemoglobinaemia: cytochrome b_5_ reductase deficiency. Br J Haematol.

[ref_4VV3VVM2] Power Gordon G., Bragg Shannon L., Oshiro Bryan T., Dejam Andre, Hunter Christian J., Blood Arlin B. (2007). A novel method of measuring reduction of nitrite-induced methemoglobin applied to fetal and adult blood of humans and sheep. J Appl Physiol (1985).

[ref_TK94U82B] Rockwood Gary A., Armstrong Kevin R., Baskin Steven I. (2003). Species comparison of methemoglobin reductase. Exp Biol Med (Maywood).

[ref_2XARTJWB] Seidel A. (2005). Ermittlung von Quellen für das Vorkommen von Nitro-/Aminoaromaten im Urin von Nichtrauchern.

[ref_62249YTN] Smith Roger P., Klaassen Curtis D., Amdur Mary O., Doull John (1986). Casarett and Doull’s toxicology. The basic science of poisons.

[ref_KYWFGZ5C] Souza Marília Cristina Oliveira, González Neus, Rovira Joaquim, Herrero Marta, Marquès Montse, Nadal Martí, Barbosa Fernando, Domingo José Luis (2023). Assessment of urinary aromatic amines in Brazilian pregnant women and association with DNA damage: influence of genetic diversity, lifestyle, and environmental and socioeconomic factors. Environ Pollut.

[ref_BQGFHTNG] Tabacova S., Balabaeva L., Little R. E. (1997). Maternal exposure to exogenous nitrogen compounds and complications of pregnancy. Arch Environ Health.

[ref_XXJWBH69] Tabacova S., Baird D. D., Balabaeva L. (1998). Exposure to oxidized nitrogen: lipid peroxidation and neonatal health risk. Arch Environ Health.

[ref_3C4AWHCS] Wright R. O., Lewander W. J., Woolf A. D. (1999). Methemoglobinemia: etiology, pharmacology, and clinical management. Ann Emerg Med.

